# An observation tool for instructor and student behaviors to measure in-class learner engagement: a validation study

**DOI:** 10.3402/meo.v19.24037

**Published:** 2014-10-10

**Authors:** Mustafa K. Alimoglu, Didar B. Sarac, Derya Alparslan, Ayse A. Karakas, Levent Altintas

**Affiliations:** 1 Department of Medical Education, Faculty of Medicine, Akdeniz University, Antalya, Turkey; 2 Faculty of Medicine, Akdeniz University, Antalya, Turkey; 3 Department of Dermatology, Faculty of Medicine, Akdeniz University, Antalya, Turkey; 4 Department of Medical Education, Faculty of Medicine, Kocaeli University, Kocaeli, Turkey

**Keywords:** student involvement, quantity, teaching methods

## Abstract

**Background:**

Efforts are made to enhance in-class learner engagement because it stimulates and enhances learning. However, it is not easy to quantify learner engagement. This study aimed to develop and validate an observation tool for instructor and student behaviors to determine and compare in-class learner engagement levels in four different class types delivered by the same instructor.

**Methods:**

Observer pairs observed instructor and student behaviors during lectures in large class (LLC, *n*=2) with third-year medical students, lectures in small class (LSC, *n*=6) and case-based teaching sessions (CBT, *n*=4) with fifth-year students, and problem-based learning (PBL) sessions (~7 hours) with second-year students. The observation tool was a revised form of STROBE, an instrument for recording behaviors of an instructor and four randomly selected students as snapshots for 5-min cycles. Instructor and student behaviors were scored 1–5 on this tool named ‘in-class engagement measure (IEM)’. The IEM scores were parallel to the degree of behavior's contribution to active student engagement, so higher scores were associated with more in-class learner engagement. Additionally, the number of questions asked by the instructor and students were recorded. A total of 203 5-min observations were performed (LLC 20, LSC 85, CBT 50, and PBL 48).

**Results:**

Interobserver agreement on instructor and student behaviors was 93.7% (κ=0.87) and 80.6% (κ=0.71), respectively. Higher median IEM scores were found in student-centered and problem-oriented methods such as CBT and PBL. A moderate correlation was found between instructor and student behaviors (*r*=0.689).

**Conclusions:**

This study provides some evidence for validity of the IEM scores as a measure of student engagement in different class types.

Educational theories and empirical data suggest that learning should be an active process. The learner should construct his/her own understanding and link new information to existing knowledge. According to social constructivist theory, learning can be better achieved by social interactions in the learning environment (1, 2). Active learning strategies fostering the students to interact with each other and the instructor via discussions, talks, questions, etc., may yield desirable learning outcomes in terms of knowledge, skills, or attitudes (3, 4). In-class learner engagement as an important keystone of active learning strategies is known to stimulate and enhance the learner's assimilation of the content and concepts (5–7). It is not easy to quantify in-class learner engagement although its value is well-appreciated and efforts are made to enhance it.

The literature on classroom climate began to grow in the 1950s and 1960s, with much attention to assessing teacher behavior and classroom interaction (8). Two primary methods of assessing student engagement described in the educational literature include direct observation and student self-report. Because students’ abilities to assess their own behaviors are questioned and self-report requires student time for completion, direct observation is preferable (9–12).

Three previously published observation instruments called ‘Stallings Observational System’ (SOS), ‘Attending Round Observation System’ (AROS), and ‘Code for Instructional Structure and Student Academic Response’ (CISSAR) had promising effects to develop observation measures (12–14). Both the SOS and the CISSAR were designed for elementary and secondary education settings and involve recording teachers’ and students’ overt behaviors for short periods at specified time intervals. The AROS was designed for medical education, but it only documents behaviors of teachers, not students. Following principles and keeping some items of these three instruments, O'Malley et al. developed and validated an instrument called STROBE to assess in-class learner engagement in health professions settings (15). The STROBE is an observation document to record details of the learning environment, types and direction of instructors, and student behaviors. In 5-min cycles, observers circle the best fitting items on the form for observed instructor and student behaviors.

In our school of medicine, a hybrid curriculum (mix of integrated courses and problem-based learning [PBL] modules) is implemented. PBL is applied in preclinical years as the student-centered pedagogy. In clinical years, some student-centered and problem-oriented methods such as case discussions and patient management problems take place in the program. There are also substantial amount of lectures delivered in large- or small-size classrooms. Because of diversity in the program and pedagogies, faculty and students undertake different roles and responsibilities in different learning environments. Such environments require different levels of in-class student engagement, for example, students are expected to be more active in the PBL sessions but less in lectures. However, we do not have any concrete evidence beyond personal observation and assumptions to confirm that requirements of different learning environments in terms of student engagement are actually met. Additionally, clinical teachers in our institution are trying some experimental methodologies to increase student involvement and active participation in their classes. Again, there is no evidence whether such methods really support in-class student engagement especially when the difference in learner engagement in experimental and former methods is not clearly observable. The STROBE seems to be a promising tool to gather data on this purpose.

The purpose of this study was to develop and validate an observation tool to determine and compare in-class learner engagement levels via observing instructor and student behaviors in four different class types delivered by the same instructor.

## Methods

### Observed class types, instructor, and student characteristics

Four types of classes delivered by the same instructor from the Department of Dermatology were observed in the same academic year. The instructor had 10 years of teaching experience in medical education. She was very keen on improving her teaching skills and trying new approaches in classes. Student feedback scores on her teaching activities were satisfactorily high (mean 4.34 over 5 for the last 5 years).

Observed class types are as follows:

Lecture in large classroom (LLC): This was a typical lecture performed in a large classroom with participation of around 100 students and one instructor. The instructor gave a presentation using PowerPoint slides and interacted with the students rarely. The title of the lecture that we followed was ‘physical examination of the skin’, which composed of two sessions (90 min) delivered to third-year medical students.

Lecture in small classroom (LSC): This was also a typical lecture performed in a small size classroom with participation of 21–29 students and one instructor. The instructor presented the content using slides and drawings on the board. Although the main teaching style was a heavily one-sided presentation, the instructor asked some questions and started some discussions to create an interactive learning environment throughout the session. The title of the class was ‘dermatitis’, which composed of three sessions (105 min) delivered to fifth-year medical students in clinical clerkships. This set was observed two times (210 min).

Case-based teaching (CBT): The instructor created the class design and flow. The title of the class was ‘pruritic patient management’ and was delivered to the same fifth-year medical students who had participated in above mentioned LSCs, with 1 week interval. Duration of the class was 90 min. The class was heavily patient problem oriented and the main pedagogy was problem solving. There were three phases in the class duration. In the first phase, the instructor showed some dermatologic lesions on slides and asked some questions to the entire class about the diagnosis of these lesions. In the second phase, the instructor divided the class into three groups and delivered a different poster to each group. On posters, there were some lesion pictures, patient information, and assignments for group work such as making diagnosis and differential diagnosis, creating a treatment plan, or classifying a disease group, etc. The groups wrote their answers on posters and interchanged the posters each 5 min. At the end of a 15-min period, a representative from each group presented the results of their group work on three posters. The instructor gave feedback to each group and provided some additional information if necessary. In the final phase, the instructor asked the class to draw the microscopic view of common microbial agents leading dermatologic diseases and voluntary students performed this on the writing board. In summary, the class started with assignments (recognize, diagnose) for the entire class, and went on with assignments for subgroups (analyze, solve), and ended with assignments (create) for individuals. Complexity of the assignments gradually increased during the process.

This set was observed two times (180 min) in the current study.

PBL session: These sessions were performed in small groups with participation of 9–10 students and one tutor. Each session took nearly a half day. The learning stimulant was a written scenario simulating the real-life patient problems. Patient details were progressively disclosed throughout the session and learners analyzed the newly disclosed information to identify the important facts and deficiencies in their knowledge that were needed to solve the case. The expected role of the tutor was providing guidance by asking questions and starting discussions only when needed. Between the sessions, learners addressed these knowledge deficiencies and came to the next session prepared to apply their new knowledge. In our school PBL is implemented in the first 2 years of medical education within a hybrid program, a mix of PBL and integrated curriculum. There are five 1-week PBL modules in each year and each module consists of three half-day discussion sessions. The PBL group observed in this study was composed of nine second-year medical students who had 27 sessions of experience in the past. The disease held in the scenario was a common health problem (Pneumonia). The group was observed more than 7 hours in total.

Observed class types and characteristics are outlined in Table 1.

**Table 1:** Observed class types and characteristics

Class type	Number of 5-min cycles	Content	Year	Number of students
LLC	20	Physical examination of the skin	3rd	104–110
LSC	85	Dermatitis	5th	21–29
CBT	50	Pruritic patient management	5th	24–29
PBL	48	Pneumonia	2nd	9

LLC=lecture in large class; LSC=lecture in small class; CBT=case-based teaching; PBL=problem-based learning.

Observation tool: A revised and extended form of STROBE (15) was used to observe and record behaviors of the instructor and students in classes. Revision was made protecting the main statements and principles of the original STROBE. While revising the form, we focused on ‘active student engagement’ and discussed which kind of instructor and student behaviors are effective on ‘active student engagement’.

Consequently, we created a list of instructor and student behaviors to have student and instructor behavior scales (five items scored 1–5 in each). The first two items of the student behavior list were about non-participating personal behaviors without any communication and the remaining three items (2–5) were about gradually increasing levels of communication with the instructor and other learners. Similarly, behaviors in the instructor list were sorted out from teacher-oriented to learner-oriented instructor actions. Each item was defined as follows:

*Instructor behavior scale:*

The instructor is

1. talking to entire class while all the students are passive receivers (1 point)
2. telling/asking one or a group of students, or teaching/showing an application on a student (e.g., a physical examination or history taking method) while the rest of the class is listening or following (2 points)
3. starting or conducting a discussion open to whole class, or assigning some students for some learning tasks (e.g., creating student groups to discuss different aspects of the subject matter) (3 points)
4. listening/monitoring active discussion with one or a group of students (4 points)
5. listening/monitoring active discussion with entire class (5 points)

*Student behavior scale:*

Student is

1. engaged with non-educational material such as mobile phone, hand bag etc.; browsing a book, notes etc.; whispering to a friend (1 point)
2. reading or writing something (maybe following the lecture from a published material or taking notes) (2 points)
3. listening to the instructor or a talking student/looking at slides or board (3 points)
4. talking to the instructor (questioning, answering, discussing, etc.), reading something (e.g., a patient script) to entire class or writing something (e.g., major signs of a disease) on the board, flip-chart etc. (4 points)
5. talking/discussing (asking, answering, explaining, etc.) with one or a group of students on the subject matter (5 points)

A sample of the revised form called ‘In-class Engagement Measure (IEM)’ is provided in the Appendix.

Observation process: The observers, except for the researchers, were trained about observation procedure, description of observable behaviors, how to take position in different rooms with different groups, and how to select individuals to observe. Five observers, as pairs or threesomes, in LLC, observed and recorded the instructor and student behaviors in different classes. Two of the observers were from the research team and the remaining three were third-year medical students. Observation unit was a 5-min cycle. The cycle proceeds as follows: First, the observer writes the starting time of the cycle and information about the class (title, instructor's name, and number of students). Next, the observer selects a student from the class and observes the selected learner for 20 sec, marking the type of engagement the learner exhibits. This is performed four times with different students in succession. The observer also observes the instructor and marks the instructor's behavior. Then, for the remainder of the STROBE cycle, the observer tallies the number of questions asked by all students –not only observed ones – and the instructor. The primary purpose of this last step is to keep the observer focused until the next cycle begins. However, the number of the questions may also give an idea about learner-to-learner and learner-to-teacher interaction level that can be an indicator to show in-class learner engagement degree. Each 5-min cycle consists of four 20-sec observations of individual learners.

Observers independently selected the students, observed, and marked their behaviors separately. Depending on the number of the observers, the classroom was divided into two or three and each of the observers selected the students from their own section. They were asked not to observe the same student repeatedly if possible.

A total of 203 cycles – with 203 instructor and 812 student scores – were recorded (LLC 20, LSC 85, CBT 50, and PBL sessions 48).

For validity of the IEM, we gathered data in two ways to investigate if it really measures what it intends to measure. First, we asked for the opinions of experienced academicians in the field of medical education by face-to-face interviews or correspondence to determine if this tool has a capacity to measure in-class learner engagement accurately. We also gave an oral presentation in a national meeting, in which nationwide experts of the field of medical education came together, and had feedback from the audience (16). As the second validity measure, we planned to focus on results obtained from lectures and the PBL sessions because the difference between two teaching methods in terms of learner engagement is well known in the literature (16). We investigated the ability of the IEM to demonstrate this difference as low scores in lectures and higher scores in the PBL sessions. We hypothesized that scores should be higher in more student-centered classes such as problem-based or team-based learning (TBL) sessions expected to have higher learner engagement. Approval of this hypothesis was held as an indicator for us to show validity of the IEM.

Finally, interobserver consistency on observed behaviors were sought to have data about reliability.

Ethical approval was granted for the study from Akdeniz University Board of Ethics on Noninvasive Clinical Human Studies.

### Statistical analyses

An inter-rater reliability analysis using the Cohen's κ statistic was performed to determine consistency among observers. Descriptive statistics were used to determine frequencies and median scores of instructor and student behaviors, and median number of questions asked in different classes. Pearson correlation analysis was performed to show correlation between behavior scores of the instructor and students. *P* values of <0.05 were set for statistical significance.

## Results

Interobserver agreement ratio on instructor behavior scores was 93.7% and κ coefficient was found 0.87 (*p*=0.000, 95% CI 0.801–0.914). Observers agreed on 80.6% of the observed student behavior scores and κ coefficient was found 0.71 (*p*=0.000, 95% CI 0.507–0.783).

Most frequent IEM scores for the instructor behaviors were ‘1’ in LLC and LSC (50 and 45.9%, respectively) and ‘3’ in CBT and PBL (44.0 and 47.9%, respectively). Most frequent scores for the student behaviors were ‘2’ in LLC (52.5%), ‘3’ in LSC and CBT (50.9 and 52.5 respectively), and ‘4’ in PBL (39.6%).

Median instructor and student behavior scores in four observed class types have been presented in Table 2.

**Table 2:** Instructor and student observation scores in different class types

	LLC Median (min–max)	LSC Median (min–max)	CBT Median (min–max)	PBL Median (min–max)
Instructor	1.5 (1.0–2.0)	2.0 (1.0–5.0)	3.0 (2.0–5.0)	4.0 (2.0–5.0)
Student	2.0 (1.0–4.0)	3.0 (1.0–5.0)	4.0 (1.0–5.0)	4.0 (1.0–5.0)

LLC = lecture in large class; LSC = lecture in small class; CBT = case-based teaching; PBL = problem-based learning.

A moderate and significant correlation was found between instructor and student behavior scores (Pearson correlation analysis, *r*=0.623, *p*=0.000).

Median numbers of the questions asked by the instructor and students have been presented in Table 3.

**Table 3:** Questions asked by the instructor and students in different class types

	LLC Median (min–max)	LSC Median (min–max)	CBT Median (min–max)	PBL Median (min–max)
Instructor	1.0 (0.0–4.0)	2.0 (0.0–6.0)	2.0 (0.0–5.0)	2.0 (1.0–5.0)
Student	0.0 (0.0–1.0)	0.0 (0.0–4.0)	1.0 (0.0–5.0)	2.0 (1.0–6.0)

LLC = lecture in large class; LSC = lecture in small class; CBT = case-based teaching; PBL = problem-based learning.

## Discussion

We developed a two-dimensional tool, the IEM, to observe instructor and student behaviors in order to determine student engagement level in a learning environment.

On the basis of educational theories and empirical data, we expected higher instructor and student behavior scores and higher number of questions in PBL and CBT than those in LLC and LSC, to confirm validity of our newly structured measurement tool. Our expectation was completely met and we were convinced that the IEM measures what it intends to measure. In a previous study, Kelly et al. compared in-class learner engagement across lecture, PBL, and TBL, using the STROBE. They found more learner-to-learner and learner-to-instructor engagement in PBL and TBL than in lecture (17). In our measurement tool, it is also possible to determine learner-to-instructor or learner-to-learner engagement by counting relevant marked items. Practically, if learner-to-learner and learner-to-instructor engagement is frequent in a learning environment, the IEM will produce higher instructor and student behavior scores and if instructor-to-learner engagement is frequent, then the scores will not be so high. Thinking this way, our results seem compatible with those of the study conducted by Kelly et al.

There may be several external factors that affect in-class learner engagement such as instructor's characteristics and relations with the students, content and complexity of the course, physical conditions of the learning environment, class size, and communication possibilities (18, 19). Some student-related internal factors such as personality, satisfaction, learning style, and stress-coping ways may also influence active involvement of the students with in-class activities (20–22). Without any intervention to these factors, medical teachers may influence learner engagement and consequently learning outcomes positively by just altering their teaching approach for more student-centered methods (1–4). It is important for teachers to recognize this power in their hands to take action. When a teacher adopts and starts to implement a new methodology, if the medical school collects data by the IEM and shows that the intervention really works, nothing will be as much convincing and motivating for the teacher.

This study has several limitations. First, the results of our study cannot be generalized because all sessions observed dealt with a single content area (Dermatology) delivered in one medical school with a single teacher and limited number of students. At most, this report might be inspiring for medical schools implementing hybrid programs similar to ours. The second limitation is about ability of the IEM to measure the reality. This form records only observable behaviors and assumes that the observed and reality are identical, but sometimes they may differ. For example, when an observer recognizes a student sitting on his chair and looking at the instructor, he will naturally circle the item ‘listen to the instructor’, but the student may be making holiday plans in thought at that moment. Another limitation is about the study design because it focuses on just learner engagement and neglects the effect of engagement on outputs. The relation between in-class learner engagement and some outputs such as student satisfaction, academic achievement, or retention of knowledge should be investigated in future studies.

## Conclusions

This study provides some evidence for validity of the IEM scores as a measure of student engagement in different class types. Student-centered and problem-oriented methods with less instructor input and more student involvement such as PBL and CBT are associated with enhanced in-class learner engagement.

## Appendix

 In-class Engagement Measure (IEM) 5 min observation form

**Table:**

Date and hour:
Observer's name:
Class title:
Instructor's name:
Number of students:
Special notes:

**Table:**

BEHAVIORS
** Instructor**
1- Talking to entire class while all the students are passive receivers
2- Telling/asking to one or a group of students, or teaching/showing an application on a student
3- Starting or conducting a discussion open to whole class, or assigning some students for some learning tasks
4- Listening/monitoring actively discussing one or a group of students
5- Listening/monitoring actively discussing entire class
Other:

**Table:**

Student				

	Student 1	Student 2	Student 3	Student 4
Engaged with non-educational material/browsing a book/notes/whispering to a friend etc. Reading or writing something (including following the lecture from a published material or taking notes) Listening to the instructor or a talking student/looking at slides or board Talking to the instructor/reading something to entire class or writing something on the board, flipchart etc. Talking/discussing with one or a group of students on the subject matter				
Other:				

**Table:**

**Number of questions**	Student:	Instructor:

**Comments:**		
